# Innovative Approaches in Knee Osteoarthritis Treatment: A Comprehensive Review of Bone Marrow-Derived Products

**DOI:** 10.3390/biomedicines12122812

**Published:** 2024-12-11

**Authors:** José Fábio Lana, Joseph Purita, Madhan Jeyaraman, Bianca Freitas de Souza, Bruno Lima Rodrigues, Stephany Cares Huber, Carolina Caliari, Gabriel Silva Santos, Lucas Furtado da Fonseca, Ignacio Dallo, Annu Navani, Marco Antônio Percope De Andrade, Peter Albert Everts

**Affiliations:** 1 Medical School, Max Planck University Center (UniMAX), Indaiatuba 13343-060, SP, Brazil; josefabiolana@gmail.com (J.F.L.); jpurita@aol.com (J.P.); doctorignaciodallo@gmail.com (I.D.); annu@navani.net (A.N.); everts@me.com (P.A.E.); 2 Department of Orthopedics, Brazilian Institute of Regenerative Medicine (BIRM), Indaiatuba 13334-170, SP, Brazil; biancafreitas.biomed@hotmail.com; 3 Regenerative Medicine, Orthoregen International Course, Indaiatuba 13334-170, SP, Brazil; madhanjeyaraman@gmail.com (M.J.); brunolr.ioc@gmail.com (B.L.R.); stephany_huber@yahoo.com.br (S.C.H.); ffonsecalu@gmail.com (L.F.d.F.); 4 Medical School, Jaguariúna University Center (UniFAJ), Jaguariúna13911-094, SP, Brazil; 5 Clinical Research, Anna Vitória Lana Institute (IAVL), Indaiatuba 13334-170, SP, Brazil; 6 Department of Orthopedics, ACS Medical College and Hospital, Dr MGR Educational and Research Institute, Chennai 600077, Tamil Nadu, India; 7 Cell Therapy, In Situ Terapia Celular, Ribeirão Preto 14056-680, SP, Brazil; caliari.carolina@gmail.com; 8 Comprehensive Spine & Sports Center, Campbell, CA 95008, USA; 9 Department of Locomotor Apparatus, Federal University of Minas Gerais (UFMG), Belo Horizonte 31270-901, MG, Brazil; marcoapercope@gmail.com

**Keywords:** knee osteoarthritis, bone marrow aspirate, bone marrow aspirate concentrate, stem cells, regenerative medicine

## Abstract

Knee osteoarthritis (OA) is a chronic articular disease characterized by the progressive degeneration of cartilage and bone tissue, leading to the appearance of subchondral cysts, osteophyte formation, and synovial inflammation. Conventional treatments consist of non-steroidal anti-inflammatory drugs (NSAIDs), analgesics, and glucocorticoids. However, the prolonged use of these drugs causes adverse effects. NSAIDs, for instance, are known to be nephrotoxic, increasing the damage to articular cartilage. New therapies capable of accelerating the process of tissue regeneration and repair are being discussed, such as the use of orthobiologics that are naturally found in the body and obtained through minimally invasive collection and/or laboratory manipulations. Bone marrow aspirate (BMA) and bone marrow aspirate concentrate (BMAC) are both rich in hematopoietic stem cells, mesenchymal stem cells (MSCs), and growth factors (GFs) that can be used in the healing process due to their anabolic and anti-inflammatory effects. The aim of this literature review is to assess the efficacy of BMA and BMAC in the treatment of knee OA based on the favorable results that researchers have obtained with the use of both orthobiologics envisaging an accelerated healing process and the prevention of OA progression.

## 1. Introduction

**Figure 1:**
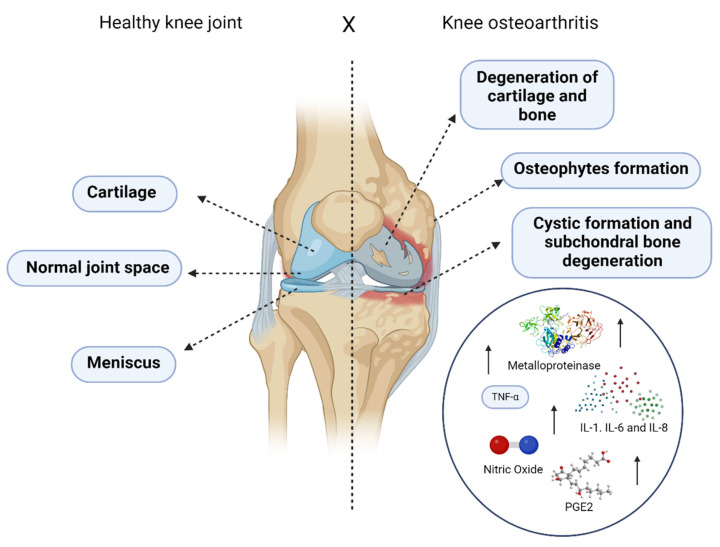
Progression and changes in cartilage and bone due to KOA and the high levels of pro-inflammatory cytokines. Created with BioRender.com, accessed on 4 March 2024.

Knee osteoarthritis (KOA) is a disease characterized by the wear of articular cartilage and bone changes and may have several causes and risk factors. Bone sclerosis, capsular fibrosis, and osteophyte formation are the results of tissue growth [1]. KOA involves abnormal remodeling driven by inflammatory mediators within the joint (Figure 1). Pathological changes in KOA include articular cartilage degradation, subchondral bone thickening, osteophyte formation, synovial inflammation, degeneration of ligaments, menisci, and joint capsule hypertrophy [2,3]. Additionally, periarticular muscles, nerves, bursae, and local fat pads are affected, contributing to KOA pathology and symptoms [2,4]. Infrapatellar fat pad (IFP) and patellar ligament thickness changes further underscore KOA as a disease of the entire joint [5]. Genetic factors, age, and a sedentary lifestyle are also risk factors for the development of KOA [6]. KOA is a major cause of musculoskeletal pain and is the most common cause of incapacity and deficiency in Western industrialized countries [7]. The symptoms are chronic pain, joint crepitus, and an inability to perform daily activities, and KOA patients commonly develop depression and other mental problems. Studies have shown that sleep disturbances are present in 12% of KOA patients, as are depression in 12% and anxiety in 7%, which are identified as factors that can impact function and capacity in KOA patients [8,9,10].

Diagnosis is based on laboratory exams, imaging exams such as magnetic resonance imaging (MRI) and X-rays, and physical exams, which are used to evaluate patients’ symptomatic complaints. Conventional treatments consist of drugs, such as non-steroidal anti-inflammatory drugs (NSAIDs) and analgesics, as well as surgeries. The prolonged use of NSAIDs and corticosteroids can interfere with the healing process and consequently affect overall joint health. Therefore, the search for new and minimally invasive treatments that can improve the patient’s quality of life, such as therapies that are capable of accelerating the process of tissue regeneration and repair, is ongoing.

Orthobiologics are widely used in orthopedics and are characterized by biological materials that can be collected and applied to patients with the potential to heal injuries more quickly [11,12,13,14]. The field of regenerative medicine employing the administration of orthobiologics, such as autologous bone marrow (BM)-derived products, has evolved significantly. BM consists of a spongy tissue located inside the central cavies of long bones and contains many different cell types, being an important source of stem cells and biomolecules [15]. This tissue can be aspirated for the use of BM-derived cells and molecular components, representing an innovative technique.

Bone marrow aspirate (BMA) and bone marrow aspirate concentrate (BMAC) are examples of these autologous products, and their use can be effective due to their anabolic and anti-inflammatory effects. They are a great source of precursor cells, such as hematopoietic and MSCs, and their associated liberation of cytokines and growth factors, which accelerate the healing process of bones and cartilage [16,17,18]. For these reasons, their use has greatly increased in studies evaluating KOA.

The main objective of this article is to evaluate the efficacy of BMA and BMAC for the treatment of knee OA through a literature review based on previously published studies. The motivation for developing this investigation is based on the favorable results that researchers have obtained with the use of both BMA and BMAC for accelerating the healing process and preventing the progression of KOA. Therefore, we believe that these autologous BM products and their associated growth factors should be thoroughly studied to establish an effective therapeutic methodology for the treatment of patients with KOA.

## 2. Methods

A literature review was conducted using the databases PubMed and Google Scholar, with searches completed up to March 2024. The aim was to assess the therapeutic potential and efficacy of bone marrow aspirate (BMA) and bone marrow aspirate concentrate (BMAC) for treating knee osteoarthritis (OA). The search was performed using a combination of the following keywords and Boolean operators: “knee”, “osteoarthritis”, “bone marrow aspirate”, “bone marrow aspirate concentrate”, “hematopoietic stem cells”, “mesenchymal stem cells”, “growth factors”, and “joint”. The search focused on human studies, with a particular emphasis on clinical trials, case reports, and comparative studies evaluating the use of BMA and BMAC in knee OA. Inclusion criteria for the review were studies that focused on the clinical application of BMA or BMAC in treating knee OA. Studies that were not related to knee osteoarthritis, animal studies, or those not published in English were excluded, unless they provided key insights applicable to human treatments. This search initially yielded a broad set of studies, which were then screened to focus on articles that specifically addressed BMA and/or BMAC use for knee OA. Studies that did not directly pertain to this focus, or were limited to other joints or conditions, were excluded. After this screening process, a total of 10 relevant articles that investigate BMA and BMAC applications for knee OA were included in this review. This review synthesized data focusing on clinical outcomes such as pain relief, functional improvement, and imaging results. The aim was to provide a comprehensive understanding of the role of BMA and BMAC in regenerative treatments for knee osteoarthritis.

### 2.1. Osteoarthritis: An Overview

Osteoarthritis (OA) affects approximately 3.3–3.6% of the global population, with around 43 million individuals experiencing moderate to severe disability, positioning it as the 11th most debilitating disease worldwide [18]. Knee osteoarthritis (KOA) is particularly common, especially among the elderly, and poses significant economic challenges due to treatment costs, modifications in lifestyle, and productivity losses [19,20,21]. In Brazil, KOA accounts for 7.5% of all work absences and is a leading reason for sickness benefits, indicating the critical need for advanced diagnostic and treatment options [22]. KOA lacks a cure, and current therapies target pain relief, range of motion, and disability reduction. Treatment choices vary based on the patient’s pain severity and functional limitations [21,23,24].

The etiology of KOA is multifactorial, involving genetic, age-related, lifestyle, and biomechanical stress factors, along with obesity, which influences joint degradation through inflammatory cytokines like adiponectin and leptin [3,25,26,27,28,29,30]. High bone density, joint trauma, and repetitive motion also contribute to the development of KOA [27,28,29]. The progression of KOA is marked by elevated proinflammatory cytokines, notably interleukin 1 (IL-1) and tumor necrosis factor-alpha (TNF-α), which drive the production of catabolic agents such as metalloproteinases (MMPs), interleukins (IL-8 and IL-6), prostaglandin E2, and nitric oxide [31,32]. MMPs degrade type II collagen, a key cause of pain in KOA, and studies indicate that they also promote angiogenesis, linking vascular growth to pain stimuli as endothelial cells invade the cartilage through degraded basal membranes [33,34,35,36].

Structural changes in KOA often extend to the subchondral bone, with edema observable via MRI, leading to persistent pain and cartilage degradation. Loss of chondrocytes and the formation of osteophytes are notable hallmarks of KOA [37,38,39]. Symptoms include joint pain, stiffness, reduced movement, muscle weakness, and impaired sleep, potentially leading to long-term disability [39]. KOA may originate in the subchondral bone, later affecting the cartilage and causing the exposure of bone tissue, joint stiffness, and significant pain [39].

The diagnosis of KOA involves clinical assessments, laboratory tests, and imaging (MRI and X-rays). The American College of Rheumatology criteria for KOA diagnosis include factors such as age over 50, pain, morning stiffness lasting for less than 30 min, and the presence of articular crepitus. ESR, rheumatoid factor levels, synovial fluid analysis, and osteophyte detection also assist in the diagnosis [40,41,42]. Symptoms like crepitus, stiffness, and joint enlargement emerge in later stages as cartilage degeneration exceeds matrix synthesis [42].

The KOA treatment focuses on mitigating joint damage and enhancing the quality of life, categorized into non-pharmacological, pharmacological, and invasive/minimally invasive options. Non-pharmacological approaches, including lifestyle modifications and exercise, have shown greater efficacy than NSAIDs and analgesics in improving patient outcomes [43,44]. NSAIDs and corticosteroids, while commonly used, pose risks such as nephrotoxicity and adverse effects on cartilage, highlighting the need for alternative therapies [30,45,46,47]. Weight loss has demonstrated benefits for pain and functionality, as well as improvements in cartilage biomarkers [48,49].

Regenerative medicine, especially orthobiologics like platelet-rich plasma, fat grafts, and bone marrow aspirate, offers promise for KOA treatment by enhancing tissue regeneration. These biologics, derived from natural body components through minimally invasive procedures, are effective in managing orthopedic injuries and accelerating healing, making them a focal point of current KOA research [50,51,52,53,54].

### 2.2. Bone Marrow Aspirate

BM is a semi-solid tissue localized in the central cavities of long and axial bones and it carries vital cellular and molecular components. The cells found in these tissues can be classified into two groups: (A) non-hematopoietic cells (pericytes, endothelial cells, osteoblasts, adipocytes, and Schwann cells) and (B) hematopoietic cells (neutrophils, lymphocytes, megakaryocytes, monocytes, and osteoclasts) [19]. BMA is the collection procedure for this tissue and involves the presence of these cells and small fragments of tissue and peripheral blood. The stimulatory effects promoted by BMA were first described in the 19th century in experiments with rabbits [20]. MSCs are a major adult stem cell population present in the BM, exerting notable physiological effects.

MSCs have the potential to differentiate into various tissues of mesenchymal origin and into different mesodermal lineages, such as osteoblasts, chondrocytes, and adipocytes [21,22]. They are also called “medicinal signaling cells” [23,24] due to their therapeutic, immunomodulatory, and trophic functions, which play crucial roles at sites of inflammation and tissue injury, including signaling and coordination of neighboring cells [25]. Due to their paracrine and autocrine effects, MSCs are also capable of modulating inflammation by increasing Treg and Th2 responses through the production of IL-10, IL-4, and IL-5 and anti-inflammatory cytokines, and reducing the activation of proinflammatory macrophage M1 phenotype whilst increasing the activity of the anti-inflammatory M2 phenotype [26,27]. A study showed that hypoxic preconditioning of MSCs isolated from the BM of the iliac crest and vertebra can increase proliferation, morphology, osteogenesis, and chondrogenesis, inhibit adipogenesis, and regulate the levels of molecular signatures of HOX genes, which are responsible for regulating the development of body segments and structures [28,29]. Hematopoietic stem cells (HSCs) are also present in the BM at lower concentrations and are adult progenitors responsible for hematopoiesis, originating from all blood cells [30]. HSCs are multi-potent cells that can proliferate through self-renewal and differentiation, have the ability to differentiate on a large scale, differentiate into cells of different lineages, and contribute directly or indirectly to the treatment of blood diseases and tissue regeneration [30,31].

**Figure 2:**
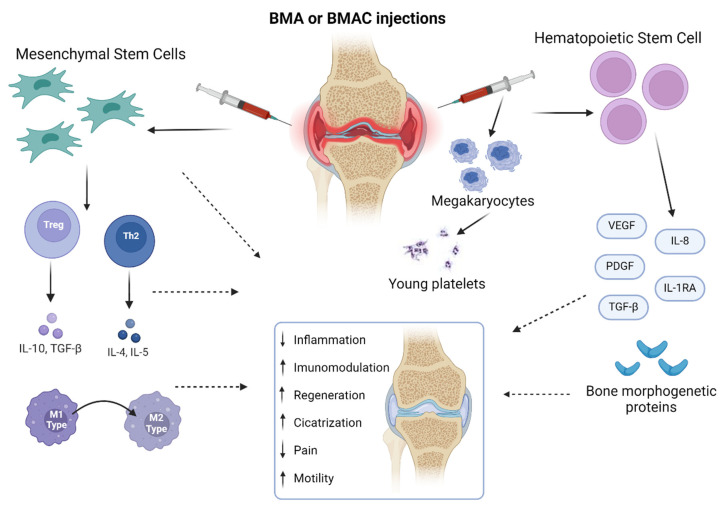
Properties of mesenchymal stem cells and hematopoietic stem cells. Created with BioRender.com, accessed on 4 March 2024.

HSCs are divided into two lineages: lymphoid and myeloid cells. The lymphoid cells are T, B, and natural killer (NK) cells, and the myeloid cells include all other cells [41]. BMA contains MSCs, HSCs, endothelial progenitors, other progenitor cells, and growth factors, including bone morphogenetic proteins (BMPs), platelet-derived growth factor (PDGF), transforming growth factor beta (TGF-β), vascular endothelial growth factor (VEGF), interleukin-8 (IL-8), and IL-1 receptor antagonists. These products are also a source of megakaryocytes that give rise to young platelets. The cells and biomolecules that are present in the BMA play a pivotal role in tissue healing and regeneration, and are widely used in orthopedics and regenerative medicine (Figure 2).

**Figure 3:**
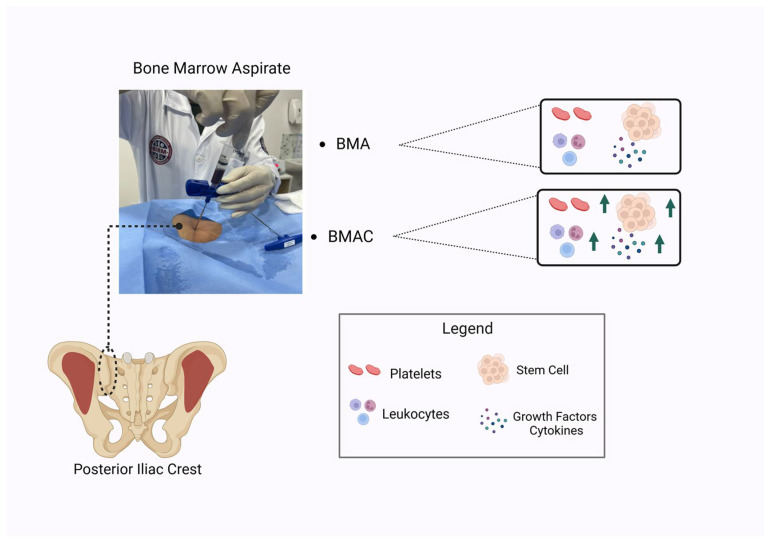
Collection procedure and BMA and BMAC components. Created with BioRender.com, accessed on 27 February 2024.

BMA is a feasible procedure in the sense that it allows for the collection and application of the biologic product without the need for laboratory manipulation, reducing the costs and maintaining regulatory compliance. The main collection site for BMA is the posterior iliac crest (Figure 3). However, the quality of the aspirate depends on the technique used for collection; the first 4–5 mL of BMA contain high-quality MSCs, and larger volumes promote the dilution of the aspirate with peripheral blood [33,34]. Another property of BMA is the formation of a clot after collection, which is also used [35]. Research has shown that BMA coagulation, even when BMA is collected with anticoagulants, can play an important role in healing due to platelet activation and degranulation, leading to cytokine and GF release in injured areas [32,36]. However, the production of these molecules is limited for PDGF, EGF, FGF, and TGF-B.

A study in mice showed myogenic differentiation for the group that received BM-derived cell injections that formed new muscle fibers, indicating that myogenic progenitors derived from BM can migrate to the injured muscle and help the regenerative process [37]. BMA can also increase the stability of the cell graft in the injured site [38]. The fibrinolytic activity of the coagulation process can lead to the release of angiogenic factors, which is essential for initiating tissue repair [39]. A study compared the effectiveness of bone healing from BMA clots using autologous bone grafts (ABGs) in rabbits with ulnar defects, and the findings were that the bone formation score increased in the group treated with BMA clots. Additionally, bone tissue formed with hypertrophic chondrocytes and calcified matrix was observed, indicating that bone repair with the BMA clot was as successful as that with the ABG [40]. Due to the clinical evidence presented, BMA has been investigated in several areas of orthopedics with the intention of promoting tissue regeneration of bones, cartilages, and soft tissues [41,42].

BMA has demonstrated significant efficacy in various clinical settings for KOA and musculoskeletal disorders, as demonstrated in several human studies. BMA injections for knee OA have shown potential therapeutic value and safety, with clinical improvements in pain and function observed over 12 months despite wide variations in cellular content [55]. BMA injections for severe knee OA resulted in significant improvements in pain, patient-reported outcomes, and walking distance over 4 years, with a 95% success [43]. Studies also indicate that the BMA matrix, by enhancing tissue repair and modulating inflammation, holds promise for regenerative medicine, although further research is needed to confirm its efficacy [38]. Comparative studies of BMA versus cortisone for glenohumeral joint OA treatment showed significant improvements in pain and function for the BMA group, though limited differences were observed in certain outcome scores [44]. Additionally, BMA injections for hip OA resulted in significant pain reduction and functional improvement at both 6 and 12 months, making it a cost-effective alternative to BMAC [45]. Collectively, these findings underscore the therapeutic potential of BMA in managing OA and enhancing tissue repair, warranting further investigation to optimize its clinical application.

Recent studies have demonstrated that BMA clot techniques are particularly valuable in diagnosing and monitoring hematological disorders like megaloblastic anemia, multiple myeloma, and chronic lymphocytic leukemia. The BMA clot analysis increases diagnostic sensitivity and allows for a morphological evaluation and an anatomopathological study comparable to bone marrow biopsy (BMB), with the advantage of not requiring decalcification. This makes it particularly effective for immunohistochemical and FISH techniques. Notably, research has shown that fracture hematoma contains higher concentrations of cytokines and growth factors compared to peripheral blood, indicating significant inflammatory and immunomodulatory properties that contribute to tissue repair.

### 2.3. Bone Marrow Aspirate Concentrate

**Figure 4:**
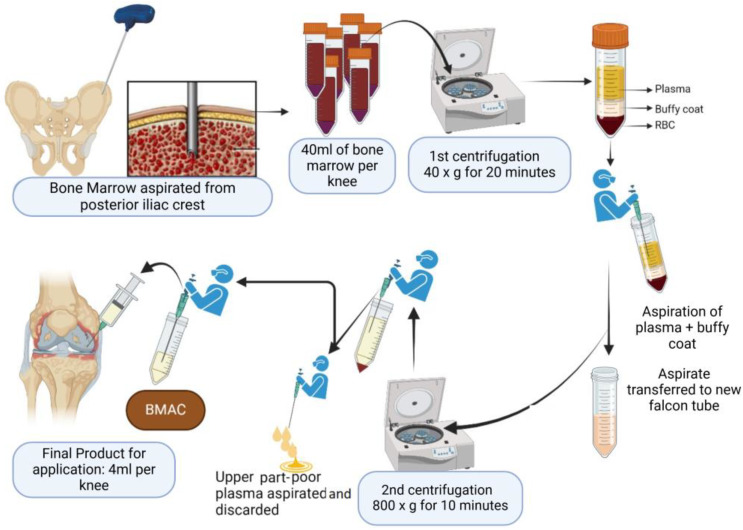
BMAC procedure in the laboratory. Created with BioRender.com, accessed on 27 February 2024.

BMAC has emerged as a new treatment for OA that is capable of promoting angiogenesis and is osteoinductive, osteoconductive, and osteogenic in nature [46,47]. As the name suggests, BMAC is basically BMA with anticoagulants subjected to a laboratory processing step, aiming to concentrate its contents [47] (Figure 4). HSCs promote cell-to-cell contact with MSCs, stimulating osteogenesis. GFs released by platelets mediate stem cell migration to the area of injury and provide adhesion sites for migrating stem cells. This product has anti-inflammatory, angiogenic, and immunomodulatory effects, increasing tissue repair due to the high levels of molecular components such as HSCs, MSCs, platelets, chemokines, and cytokines. Therefore, BMAC also plays an important role in injured tissue regeneration by replacing damaged or lost cells through the immunomodulatory action of MSCs, influencing healing through soluble factor secretion and promoting vascularization, cell proliferation, differentiation, and the modulation of the inflammatory process [47]. Studies have reported improvements in symptoms and quality of life scores for OA patients who received two to six BMAC injections at intervals of 2 to 3 months, showing the regenerative medicine potential of this autologous bioproduct [43,48,49,50]. Although BMAC needs to be manipulated in the laboratory, it is also an easy technique without the need for cell culture expansion, which reduces the issues regarding regulatory compliance [46,47]. Provided the operator works carefully in a validated facility with adequate aseptic techniques, this orthobiologic material poses no risk of disease transmission or infection; therefore, it may be used concomitantly with another procedure.

An experimental study performed with goats showed complete coverage of full-thickness chondral defects 24 weeks after the application of BMAC and hyaluronic acid [51]. In another equine model study, BMAC was applied after chondral microfactures, which, through magnetic resonance imaging and histological evaluation, successfully revealed hyaline cartilage restoration [52].

Bone marrow aspirate concentrate (BMAC) has shown promising results in treating osteoarthritis (OA) and cartilage defects across multiple studies. BMAC demonstrated significant clinical improvements in knee OA, outperforming platelet-rich plasma (PRP) and hyaluronic acid (HA) in clinical outcomes such as WOMAC and IKDC scores, with effectiveness observed from three days to twelve months [53]. Additionally, BMAC has been effective in reducing pain, enhancing activity, and decreasing reliance on pain medications, although some studies indicate no significant difference from the placebo in pain relief, suggesting the need for further research before widespread recommendation. Comparative studies of BMAC and autologous conditioned serum (ACS) for knee OA also found both treatments to be safe and effective, with BMAC showing significant improvements in WOMAC and VAS scores due to its high levels of mesenchymal stem cells (MSCs), platelets, and growth factors, which provide anti-inflammatory, angiogenic, and immunomodulatory benefits [50,54]. The BMAC efficacy in cartilage repair is supported by its ability to enhance hyaline cartilage response and modulate paracrine signaling, crucial for osteogenesis and chondrogenesis [56]. Furthermore, BMAC has shown improvements in algofunctional indices and cartilage quality, making it a viable option for delaying joint replacement in OA patients, though more extensive preclinical research and large controlled trials are necessary to fully establish its long-term efficacy and safety.

Another article described a series of knee OA patients who were treated with BMAC and concluded that BMAC injections had positive effects, including decreased pain and better functional outcomes, improving daily activities and quality of life [46,47]. BMAC injection has been successfully used for treating humeral diaphyseal fractures because it is minimally invasive and avoids possible complications associated with conventional compression plate techniques for treating humeral nonunion [57]. In knee OA, osteoarthritic chondrocytes exhibit an imbalance between anabolic and catabolic functions. This inequality leads to the degradation of the hyaline cartilage extracellular matrix, which is mediated by proinflammatory cytokines. Chronic inflammation aggravates damage to the cartilage and eventually leads to mechanical and biological dysfunction within the joint. Therefore, BMAC can aid in the treatment of knee OA because it contains a large number of GFs, including PDGF, TGF-β, VEGF, and bone morphogenetic protein (BMP)-2 and -7, which have anabolic and anti-inflammatory effects [46].

There are some differences between BMA and BMAC, such as the concentration of MSCs (Table 1). The concentration of MSCs in BMA is low, approximately from 0.01% to 0.02%; therefore, BMAC is considered to be a method for increasing the concentration of MSCs in a short time. On the other hand, BMA has greater cellular viability than BMAC because certain centrifugation parameters and manipulation techniques can relay indefinite effects on these cells [58]. The location of the collection site can also affect the number of cells; therefore, collection in the posterior iliac crest seems to be the preferred anatomical landmark because this region contains a considerable amount of biological material with a greater number of osteoblastic progenitors. The posterior iliac crest is an accessible site that is safe and facilitates BM aspiration because the patient does not usually need to be placed in discomforting positions, unless otherwise specified [17,59].

### 2.4. Implications and Challenges

BM-derived products offer potential regenerative approaches for knee OA [60]. BM-derived MSCs, for example, have the ability to differentiate into various cell types, including chondrocytes, aiding in cartilage regeneration. Additionally, bone marrow products also contain growth factors and cytokines that may further stimulate tissue repair mechanisms and reduce inflammation in the joint. However, the clinical efficacy of these treatments remains uncertain, with some studies reporting improvements in pain, function, and cartilage regeneration, while others find only modest or inconsistent benefits [61,62]. Safety concerns, including infection, immune reactions, and tumor formation, require further investigation, necessitating long-term safety data [62].

The standardization of preparation methods and quality control measures is crucial for ensuring consistency and efficacy. Variability in cell isolation, sample sizes, culture techniques, and orthobiologic preparation methods can influence outcomes, emphasizing the need for standardized protocols [63,64].

Determining the optimal dosing, frequency, and delivery methods poses challenges. Factors such as patient age, disease severity, and comorbidities must be considered. Cost-effectiveness is also a concern, given the potential expense associated with these therapies, including the cost of isolation, culture, and specialized equipment [65,66].

BM-derived products hold promise for knee OA treatment, addressing implications such as clinical efficacy, safety, standardization, optimal dosing, and cost-effectiveness is crucial for realizing their full potential in regenerative medicine. Collaborative efforts are needed to advance research and clinical practice in this field. We would also like to reiterate that although BMA and BMAC are popular orthobiologics with robust results, there is still a lack of standardization regarding the collection and processing of protocols. We previously published a manuscript proposing the “ACH” classification system [67]. A refers to BMA; C relates to BMA and BMAC; and H stands for hybrid, combining A and C. This classification delineates eight parameters that are crucial for assessing the quality of biological products. The inclusion of more parameters suggests a deeper characterization and a more intricate evaluation of the biological product in question. The ACH classification seeks to deepen the understanding of both clinical methodologies and research results, envisaging the standardization of best practices.

Recent research has identified several critical areas requiring further attention within bone marrow aspirate (BMA) applications. First, optimizing the collection technique has proven essential, with studies underscoring that variables such as aspiration site, needle gauge, aspiration speed, and volume extracted can substantially influence cell viability and overall treatment efficacy. Additionally, strategic considerations for BMA application have emerged, particularly regarding anatomical specificity; research indicates that administration at targeted locations, such as the sacral hiatus for spinal treatments, may impact broader neurological structures and potentially enhance functional recovery. Furthermore, while initial investigations employing rabbit models have shown promising effects on spinal fusion rates and osteogenic properties, a need for rigorous standardization remains. To this end, larger, randomized controlled trials are necessary to validate these preliminary findings. Finally, a focus on the development of novel BMA clot formulations has surfaced. These formulations, envisioned as osteogenic and osteoinductive three-dimensional bioscaffolds, offer the potential to obviate the need for BMA concentration or purification, while simultaneously enhancing graft site stability. The development of decellularized extracellular matrix (dECM) hydrogels has shown promise as a biomaterial scaffold that enhances the delivery and stability of BMA/BMAC therapies in regenerative applications, thus improving treatment efficacy [68].

## 3. Conclusions

BMA and BMAC are valuable autologous BM-derived products essential for tissue regeneration. These products hold promise for enhancing the healing of knee injuries, potentially expediting recovery and reducing the costs associated with surgeries and hospitalization. The growing incidence of OA, leading to increased sick leaves and early retirement, underscores the need for effective treatments. Current treatments often fall short, being either invasive or inadequate. BMA and BMAC offer orthobiologic alternatives that have shown positive effects on both cartilage and bone health in knee OA patients. Notably, knee OA often originates from pathological changes in the subchondral bone, highlighting the need for treatments that restore subchondral bone homeostasis. While BMAC can concentrate essential cells and molecules, BMA offers the convenience of being collected and applied in a single session. Despite their potential, a thorough patient evaluation is crucial to tailor treatment plans effectively. Continued research and clinical trials are necessary to refine these therapies and verify their comparative benefits.

## Figures and Tables

**Table 1:** Comparison of bone marrow aspirate (BMA) and bone marrow aspirate concentrate (BMAC).

Feature	Bone Marrow Aspirate (BMA)	Bone Marrow Aspirate Concentrate (BMAC)
Composition	Contains mesenchymal stem cells (MSCs), hematopoietic stem cells (HSCs), endothelial progenitors, other progenitor cells, growth factors (BMPs, PDGF, TGF-β, VEGF, IL-8, IL-1Ra), and megakaryocytes.	Concentrated form of BMA, containing higher concentration of MSCs, HSCs, platelets, chemokines, and cytokines.
Preparation	Collected directly from the bone marrow, typically from the posterior iliac crest, without further manipulation.	BMA subjected to laboratory processing and centrifugation to concentrate the cellular and molecular components.
MSC Concentration	Low concentration of MSCs (approximately from 0.01% to 0.02%).	Higher concentration of MSCs compared to BMA.
Cellular Viability	Greater cellular viability compared to BMAC as it is not subjected to centrifugation or manipulation.	Cellular viability may be affected by centrifugation parameters and manipulation techniques.
Regulatory Compliance	Does not require laboratory manipulation, reducing regulatory compliance issues.	Requires laboratory manipulation, necessitating regulatory compliance measures.
Coagulation	BMA forms a clot after collection, which can play a role in healing due to platelet activation and growth factor release.	Not explicitly mentioned in the provided text.
Clinical Applications	Used in orthopedics and regenerative medicine for tissue regeneration of bones, cartilage, and soft tissues.	Emerging treatment for osteoarthritis (OA) capable of promoting angiogenesis, osteoinduction, osteoconduction, and osteogenesis. Reported improvements in symptoms and quality of life scores for OA patients.
Advantages	Easy to collect and apply without laboratory manipulation, reducing costs and regulatory issues. The formed clot can aid in healing.	Higher concentration of MSCs, HSCs, and growth factors, promoting tissue regeneration, angiogenesis, and immunomodulation. Can be used concomitantly with other procedures.
Disadvantages	Lower concentration of MSCs compared to BMAC.	Requires laboratory manipulation, which may affect cellular viability and increase regulatory compliance issues.
